# A Comprehensive Pipeline for the Use of Short Read Next-Generation Sequencing (SR-NGS) in *CYP21A2* Diagnostic Genotyping

**DOI:** 10.3390/cimb48080826

**Published:** 2026-08-13

**Authors:** Irene Fylaktou, Faidon-Nikolaos Tilemis, Anny Mertzanian, Chrysi Kontse, Periklis Makrythanasis, Christina Kanaka-Gantenbein, Amalia Sertedaki

**Affiliations:** 1 Division of Endocrinology, Diabetes and Metabolism ‘Aghia Sophia’ Children’s Hospital ENDO-ERN Center for Rare Paediatric Endocrine Diseases, First Department of Pediatrics, Medical School, National and Kapodistrian University of Athens, ‘Aghia Sophia’ Children’s Hospital, 115 27 Athens, Greeceaserted@med.uoa.gr (A.S.); 2 Laboratory of Medical Genetics, Medical School, ‘Aghia Sophia’ Children’s Hospital, National and Kapodistrian University of Athens, 115 27 Athens, Greece; 3 Biomedical Research Foundation of the Academy of Athens, 115 27 Athens, Greece; 4 Department of Genetic Medicine and Development, Medical School, University of Geneva, CH-1211 Geneva 4, Switzerland

**Keywords:** *CYP21A2* genotyping, 21-hydroxylase deficiency, SR-NGS, bioinformatics pipeline, validation, congenital adrenal hyperplasia

## Abstract

**Background:** Although Short Read Next-Generation Sequencing (SR-NGS) is widely employed in diagnosis, its application in *CYP21A2* genotyping remains limited due to its high sequence homology with its pseudogene, CYP21A1P. Herein, we present (a) a complete pipeline for the diagnostic use of SR-NGS in *CYP21A2* genotyping following its assessment; (b) two distinct in-house bioinformatics pipelines for variant calling; and (c) the results by implementing this pipeline in diagnosis. **Methods**: A total of 221 subjects were studied, comprising a pilot group (*n* = 21), recruited for assessment of the assay, and a study group (*n* = 200) categorized in three subgroups, referred for *CYP21A2* genotyping. Both groups underwent SR-NGS. Two different bioinformatics algorithms for variant calling were applied and variant filtration was performed using VarAFT (v2.17). In the study group, MLPA was additionally employed. **Results:** The SR-NGS assay, employing GATK HaplotypeCaller, demonstrated 100% sensitivity and specificity when compared to Sanger Sequencing; however, complex *CYP21A2* rearrangements cannot be detected. In the study group, pathogenic variants were identified in 52.7%, 100% and 25% of cases in subgroups (a), (b) and (c) respectively, whereas gene duplications accounted for 12.3% (7/57) of subjects tested. **Conclusions**: This study provides a comprehensive protocol for the use of SR-NGS in a *CYP21A2* diagnostic genotyping, integrating complementary bioinformatics pipelines and MLPA for copy number analysis.

## 1. Introduction

21-hydroxylase deficiency (21-OHD) represents the most common form of congenital adrenal hyperplasia (CAH), exhibiting a recessive mode of inheritance. The phenotypic spectrum of 21-OHD ranges from mild (non-classic, NC) to severe (classic, C), and the genetic background is attributed to *CYP21A2* gene pathogenic variants leading to impairment in the enzymatic activity of 21-hydroxylase.

The *CYP21A2* gene resides on chromosome 6 (6p21.31), within the major histocompatibility complex (MHC) class III region, in a locus known as the RCCX fragment [1,2]. Approximately 30 Kb apart from the *CYP21A2* gene resides its highly homologous pseudogene, CYP21A1P (98% homologous in exons and approximately 96% in introns) [3]. Most pathogenic variants (75%) in the *CYP21A2* gene derive from its pseudogene, due to gene conversion events. Gene deletions, CYP21A1P-CYP21A2 chimeras, as well as de novo aberrations have also been described [4,5].

The high homology of the *CYP21A2* gene with its pseudogene renders the molecular analysis of the *CYP21A2* gene challenging. The application of SR-NGS for the analysis of *CYP21A2* has not been widely applied due to the high sequence homology with its pseudogene, which can cause read misalignment and result in the detection of variants in the CYP21A1P pseudogene (false-positive) or a failure to identify pathogenic variants in *CYP21A2* gene (false-negative) [6,7,8]. The European Molecular Genetics Quality Network (EMQN) best practice guidelines suggest the use of PCR-based sequencing and Multiplex Ligation-dependent Probe Amplification (MLPA) for the molecular investigation of the *CYP21A2* gene [6]. Although SR-NGS is not recommended for *CYP21A2* genotyping, several assays combining long-range PCR for the amplification of the *CYP21A2* gene followed by SR-NGS have been reported [9,10,11,12,13].

This study provides a complete pipeline for the use of SR-NGS in diagnostic *CYP21A2* genotyping after validating one of the first used NGS assays [9], describing two different bioinformatics pipelines for variant calling, and presents the results of 200 samples referred to our center with clinical suspicion of 21-OHD by implementing this assay in diagnostic genetic testing.

## 2. Materials and Methods

### 2.1. Subjects

A total of 221 subjects were included in this study and categorized into two groups. The first group (pilot group), recruited for the validation of the SR-NGS assay, consisted of 21 subjects whose DNA had been previously analyzed by Sanger Sequencing and MLPA. The methodology applied has been previously described [14]. The selection of subjects was based on 4 criteria: (a) representation of the most common pathogenic variants in the *CYP21A2* gene, (b) inclusion of all possible genotypes in 21-OHD (compound heterozygotes, homozygotes, heterozygotes and negatively genotyped cases), (c) subjects carrying the p.Ile173Asn pathogenic variant (in heterozygosity, compound heterozygosity and homozygosity), since this variant frequently escapes diagnosis [6], and (d) samples harboring pathogenic variants along with duplication of the *CYP21A2* gene in order to identify any differences in the frequency of the reported variants. The genotypes of the subjects in the pilot group are presented in Table 1.

The second group (study group) consisted of 200 subjects (148 females and 52 males) who were referred for *CYP21A2* genotyping during a period of 16 months (December 2023 to April 2025). This group was subcategorized according to the reason of referral. The first subgroup (a) included subjects (182/200) with CAH-related symptoms or laboratory findings or family history. Subjects with CAH-related symptoms were clinically and hormonally assessed by their pediatrician/endocrinologist. The clinical assessment was based on CAH-indicative clinical features, according to the age of presentation that had been previously reported [14]. The hormonal assessment included basal 17-hydroxyprogesterone (17-OHP), cortisol, Δ4-Androstenedione, testosterone, dehydroepiandrosterone sulfate, and adrenocorticotropic hormone (ACTH) levels, and a ACTH stimulation test.

The second subgroup (b) included subjects with a clinical diagnosis of classic 21-OHD (2/200 cases) but not a genetic diagnosis, and the third subgroup (c) consisted of subjects referred to our center for preconception carrier screening (16/200 cases). The median age of subgroup (a) was 11.7 years (1 month to 72 years) and of subgroup (c) was 38 years old (33 years to 59 years). The subgroup (b) included 2 patients with a median age of 40 years old.

Informed consent was obtained from all subjects or their legal guardians for the molecular analysis of the *CYP21A2* gene and the use of the resulting data for scientific purposes. The study has been approved by the ethical committee of the “Aghia Sophia” Children’s Hospital.

### 2.2. DNA Extraction

Genomic DNA from all subjects and their parents (when available) was isolated from peripheral blood samples with the use of QIAamp DNA Blood Mini Kit (Qiagen, Hilden, Germany) according to the manufacturer’s instructions.

### 2.3. Molecular Analysis of the CYP21A2 Gene Employing SR-NGS

**Figure 1:**
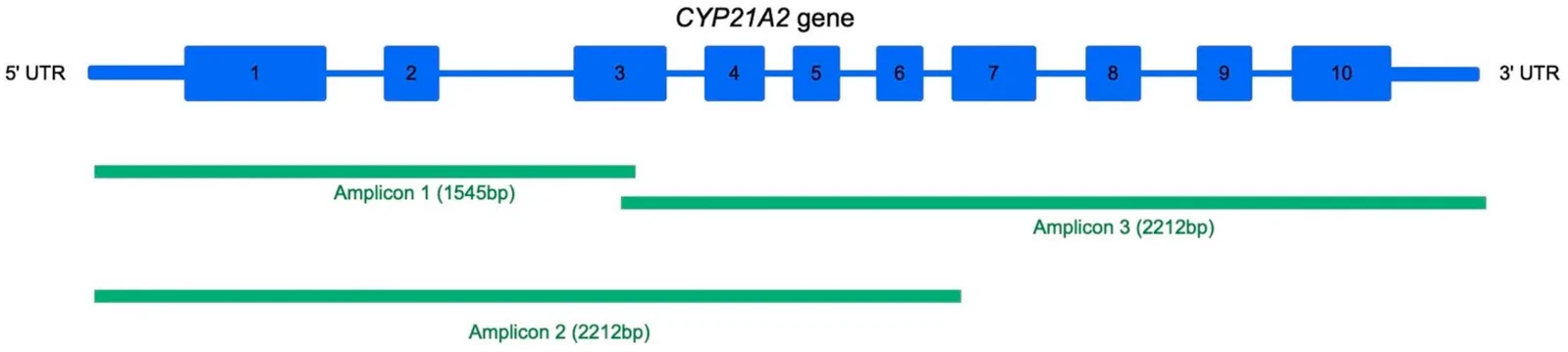
Schematic representation of the *CYP21A2* gene and the designed amplicons.

Two different approaches were applied for the analysis of the pilot group. In both approaches, the design covered the entire *CYP21A2* gene (NM_000500.9), including the 5′ and 3′ UTR (length: 3712 bp). The same previously reported amplicons have been used in SR-NGS and Sanger sequencing [15]. In Appendix A, the sequences of primers used for each amplicon are provided. In the first approach, PCR amplification of the *CYP21A2* gene was carried out using three amplicons; two amplicons of 2212 bp and one amplicon of 1545 bp (Figure 1). The first amplicon (1545 bp) included the 5′ UTR until exon 3 (in the locus where the 8 bp deletion is present in the pseudogene-p.Gly111ValfsTer21). The second amplicon (2212 bp) included the 5′ UTR until exon 6 (in the locus where the cluster of three pathogenic missense variants is present in the pseudogene-p.[Ile237Asn;Val238Glu;Met240Lys]). The third amplicon (2212 bp) covered exon 3 (in the locus where the 8 bp deletion is present in the pseudogene-p.Gly111ValfsTer21) until the 3′ UTR of the *CYP21A2* gene. This approach was carried out in all 21 samples of the pilot study. In the second approach, amplification of the *CYP21A2* gene was carried out with the use of the second and third amplicon. In the second approach, 3 samples of the pilot study were selected (samples 5, 15 and 16) in order to determine: (a) if the use of two amplicons would be sufficient to cover the entire *CYP21A2* gene, and (b) if there would be any discrepancies in the detection and frequency of all variants detected when comparing the two above-mentioned approaches.

In the study group, the second approach was employed, resulting in the amplification of the second and the third amplicons for the molecular investigation of the *CYP21A2* gene.

**Figure 2:**
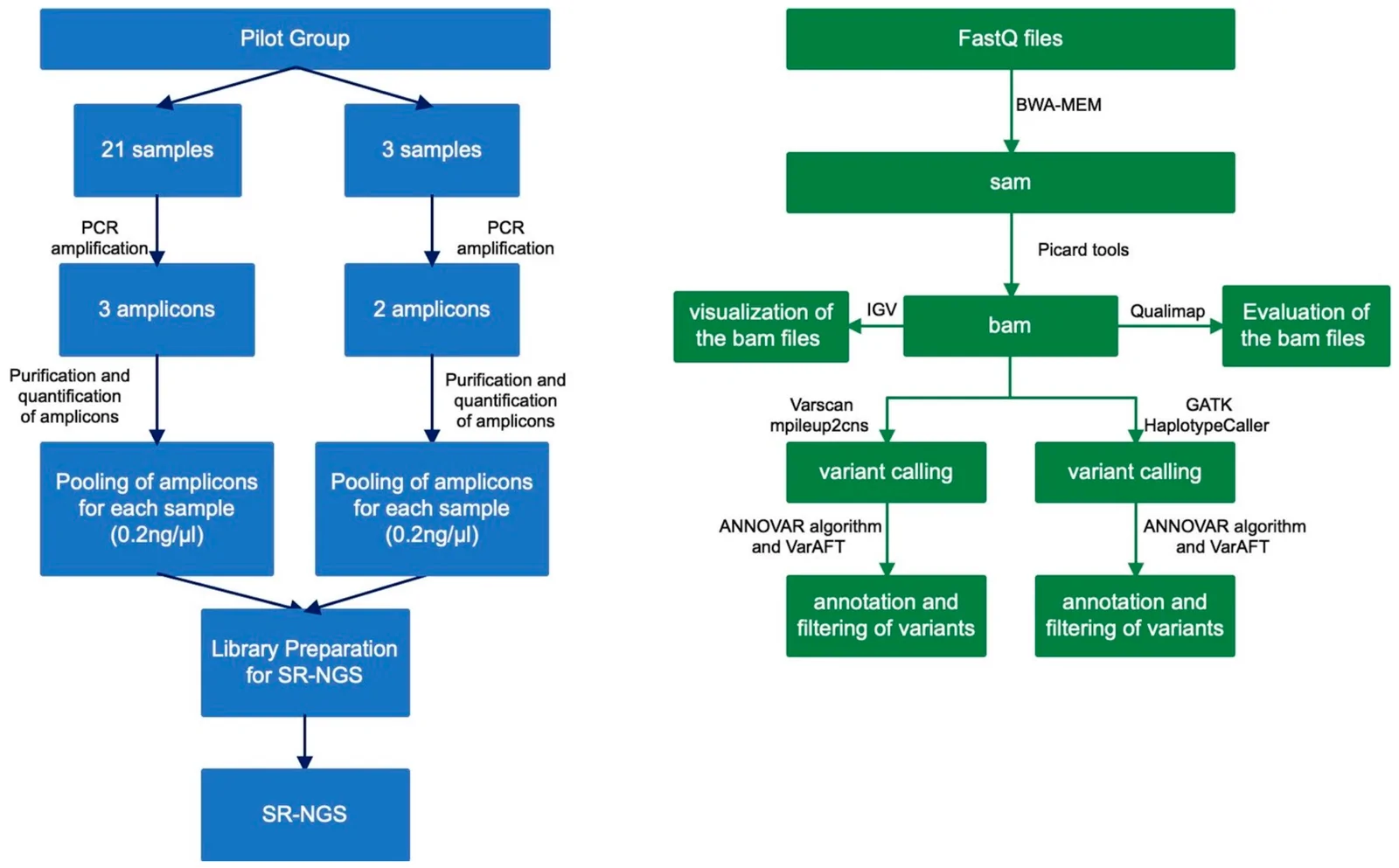
Flow chart of the SR-NGS procedure and the bioinformatics pipeline of the pilot study.

In both approaches, the PCR amplicons were purified using the Nucleospin Gel and PCR Clean-up kit (MACHEREY-NAGEL GmbH & Co. KG, Düren, Germany). The purified amplicons were quantified using the Qubit dsDNA High Sensitivity kit (Thermo Fisher Scientific Inc., Waltham, MA, USA) in a Qubit 3.0 Fluorometer. For each sample, the purified amplicons were then pooled into a final concentration of 0.2 ng/μL. All samples underwent SR-NGS, utilizing the Nextera XT DNA Library Preparation kit (Illumina, Inc., San Diego, CA, USA) and the IDT for Illumina Nextera DNA UD Indexes (96 samples) (Illumina, Inc., San Diego, CA, USA). The samples were loaded on a MiSeq Sequencing System (Illumina, Inc., San Diego, CA, USA) using the Nano Kit v2 (300-cycles) Reagent. In the pilot group, the final library consisted of a total number of 24 samples (21 samples using the first approach and 3 samples with the second approach). In Figure 2, the SR-NGS procedure for the pilot group is presented. In the study group, each run consisted of 20–24 samples.

When more than one pathogenic variant was identified in a subject of the study group, parental samples were analyzed by Sanger sequencing in order to elucidate the segregation of the pathogenic variants.

### 2.4. MLPA

**Figure 3:**
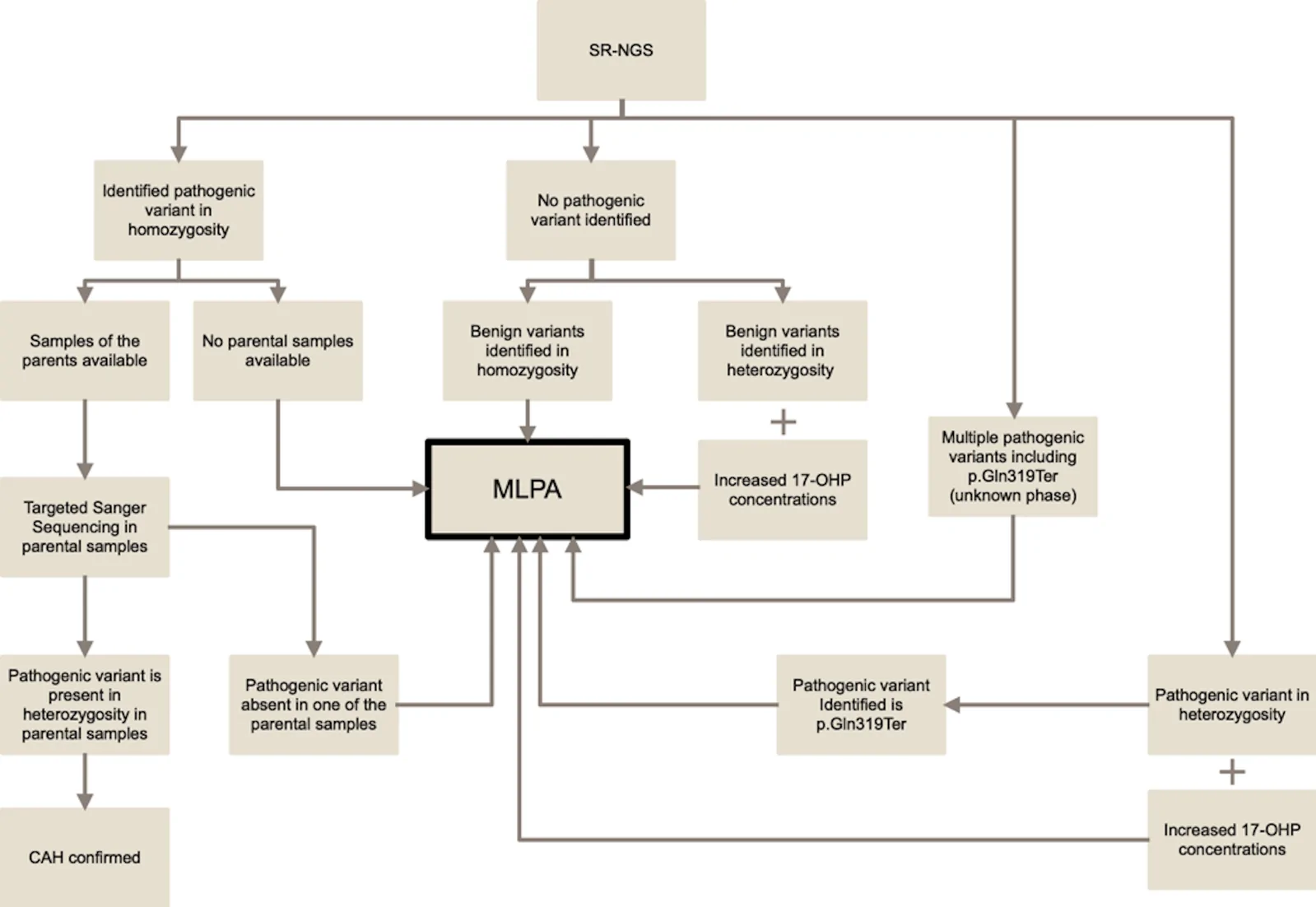
MLPA workflow of the study group after SR-NGS analysis. The (+) symbol indicates that both conditions should apply in order to proceed with MLPA.

MLPA kit P050-D1 CAH (MRC Holland, Amsterdam, The Netherlands) was used for the detection of aberrations in the copy number of the *CYP21A2* gene. MLPA was employed in the study group’s DNA samples after SR-NGS was performed. The selection of samples undergoing MLPA was based on one of the following requirements: (a) the identification of the p.Gln319Ter in heterozygosity that could be indicative for duplication of the *CYP21A2* gene; (b) the absence of any heterozygosity in *CYP21A2* gene, since this might be indicative for a possible deletion of the *CYP21A2* gene; (c) the presence of a pathogenic variant in homozygosity (when parental samples were not available or when one of the parental samples did not harbor the pathogenic variant of his offspring), indicating deletion of the *CYP21A2* gene and (d) cases where the laboratory findings (increased 17-OHP concentrations) were not in concordance with the results obtained from the SR-NGS (Figure 3). From the 200 samples, 57 samples [45 from subgroup (a), 2 from subgroup (b) and 10 from subgroup (c)] underwent MLPA, of which 37 had no identified pathogenic variant, 9 were heterozygous for p.Gln319Ter, 7 were homozygous for a pathogenic variant, 1 had no pathogenic variant identified but all the identified benign variants were in homozygosity, and 3 had laboratory findings (increased 17-OHP concentrations) inconsistent with the results obtained after SR-NGS.

The MLPA analysis was performed with the use of the Coffalyser.Net software (v.220513.1739 and v.240129.1959) with thresholds defined according to the software’s instructions.

Cases with *CYP21A2* duplication (with or without the p.Gln319Ter) were managed as previously described [14].

### 2.5. Bioinformatics Pipeline for the Analysis of SR-NGS Data in the CYP21A2 Gene

SR-NGS raw data generated by the MiSeq Sequencing System were analyzed, implementing an in-house bioinformatic pipeline. Reads were aligned exclusively to the *CYP21A2* gene reference sequence (GRCh37/hg19) using the Burrows–Wheeler Aligner (BWA-MEM; v.0.7.18-r1243) algorithm [16], after “masking” the *CYP21A1P* pseudogene from the reference FASTA file. Default alignment parameters were applied, including a minimum seed length of 19 bp and a mismatch penalty optimized for Illumina reads, with output generated in the Sequence Alignment Map (SAM) format. The resulting SAM files were converted to BAM files and sorted with Picard tools (v.3.3.0). PCR and optical duplicates were identified and marked using the Picard MarkDuplicates algorithm (http://broadinstitute.github.io/picard/, 1 November 2024), base quality score recalibration (BQSR) was performed using the Genome Analysis Toolkit (GATK v.4.6.1.0) [17], and local realignment around indels was conducted to minimize alignment artifacts in regions containing insertions or deletions, improving variant-calling accuracy. Quality control metrics, including mean coverage depth, coverage uniformity, mapping quality, and duplication rates, were assessed using Qualimap, while read-level inspection and variant visualization were performed using Integrative Genomics Viewer (IGV) [18,19].

Subsequently, variants were independently called using two different algorithms to compare their analytical performance and maximize variant detection. GATK HaplotypeCaller (v.4.6.1.0) was used with the default parameters, whereas VarScan mpileup2cns (v.2.4.6) was applied to pileup files generated from the aligned BAM files using the SAMtools (v.1.22.1) mpileup function [20]. Unlike GATK, which operates directly on BAM files, VarScan requires a large intermediate pileup file summarizing the aligned sequencing reads at each genomic position prior to variant calling. Variant calls generated by the two algorithms were compared during pipeline validation, and discrepant calls were resolved by manual inspection of the corresponding BAM files using the Integrative Genomics Viewer (IGV). Variant annotation was performed using ANNOVAR (v.2018-04-16) [21]. Functional effects were annotated according to RefSeq gene models, and population allele frequencies were obtained from public databases (e.g., gnomAD). Variant filtering was performed with the VarAFT v2.17 (http://varaft.eu, 1 February 2022) application (Figure 2) [22]. Variants were retained if they met the following criteria: minimum read depth ≥15×, variant allele frequency (allelic balance) ≥10% for heterozygous calls, base quality ≥ 20, and mapping quality ≥ 30. Final variant classification was evaluated according to the recommendations of the American College of Medical Genetics and Genomics (ACMG) [23].

### 2.6. Assessment of the SR-NGS Technique in Pilot Group’s Samples

Assessment of the SR-NGS technique was based on metrics recommended for NGS [24]. These metrics include specificity, sensitivity, adn reproducibility/robustness. All of these metrics were calculated only for SR-NGS when compared with Sanger sequencing. Thus, deletions and duplications of the *CYP21A2* gene were not evaluated. Specificity and sensitivity were calculated using the Clopper–Pearson exact binomial method, with 95% confidence intervals [25]. Specificity and sensitivity were calculated at the base level for each sample and pooled across all samples for the analysis performed with VarScan mpileup2cns and GATK HaplotypeCaller. All statistical analyses were performed in R studio (v.1.2.5033). For each sample, True Positives (TP), True Negatives (TN), False Positives (FP) and False Negatives (FN) were calculated. Variants identified in the vcf and BAM files were considered as TP, variants identified in the bam file and not found in the vcf file or variants that were present in Sanger sequencing and not present in the vcf files were considered as FN. Variants that were found in the vcf file but not in the sequence of the Sanger sequencing were considered as FP. Variants representing the reference variants were considered as TN. For the calculation of TN, the length of the *CYP21A2* design was used (length: 3712 bp). Loci that were not covered by Sanger sequencing (such as the 5′ of *CYP21A2*) were excluded from analysis and, as a result, they were not calculated in TN. Hence, variants detected through SR-NGS that could not be verified by Sanger sequencing, due to the inability of the latter to cover these regions, were also excluded from the assessment of the SR-NGS assay.

To assess the reproducibility (between run-variability) and the repeatability (within-run variability) of the SR-NGS assay, a positive reference sample was used in every run of the study group, and one sample (single library) was used twice in a single run, respectively. To assess the assay’s robustness, the QC metrics of the pilot run were used.

All variants identified in the samples of the pilot group were compared to Sanger sequencing (gold standard technique) results.

Variant allele fraction thresholds were also set. Allele fractions ranging from 0.1 to 0.96 were considered as heterozygous, while allele fractions > 0.97 were homozygous. Variants identified with an allele frequency < 0.1 were not considered as true positive variants and were excluded from the validation of the SR-NGS assay.

### 2.7. Cost Estimation

To determine the cost for Sanger sequencing as well as SR-NGS, prices were estimated based on the distributor’s 2024 price list. The Sanger sequencing cost estimation was based on the cost of the enzymes used for PCR purification, the kit for the sequencing reaction, the POP-7 polymer, the formamide and the consumables (plates and septa) for the ABI-3500 sequencing system (Life Technologies Europe BV, Bliswijk, Netherlands).

The calculation of the cost for SR-NGS was based on the kits used for PCR purification and quantification, the kit for the Library preparation, the indexes, the reagent for the MiSeq Sequencing System and the consumables used (plates and PCR films).

Taxes were not included in the cost calculation.

## 3. Results

### 3.1. Metrics of the Run in the Pilot Group and in the Study Group

The mean coverage of the pilot group’s samples was 4525 (range: 3424–5565) reads. The cluster density of the run was 1258 K/mm^2^, the Cluster Passing Filter was 92.9% and the ≥Q30 was 92.9%. The mean of the mapped reads was 99.28% (98.54–99.69%).

In the study group, the mean coverage of all samples in the exonic regions of the *CYP21A2* gene was 5118 (range:2692–7587) reads, and the mean of the mapped reads was 99.07% (range: 95.76–99.64). All runs performed in the study group had a cluster density between 1100 K/mm^2^ and 1315 K/mm^2^, Cluster Passing Filter > 88.2%, and the ≥Q30 was >92.1%.

### 3.2. Assessment of the SR-NGS Technique in the Pilot Group

**Figure 4:**
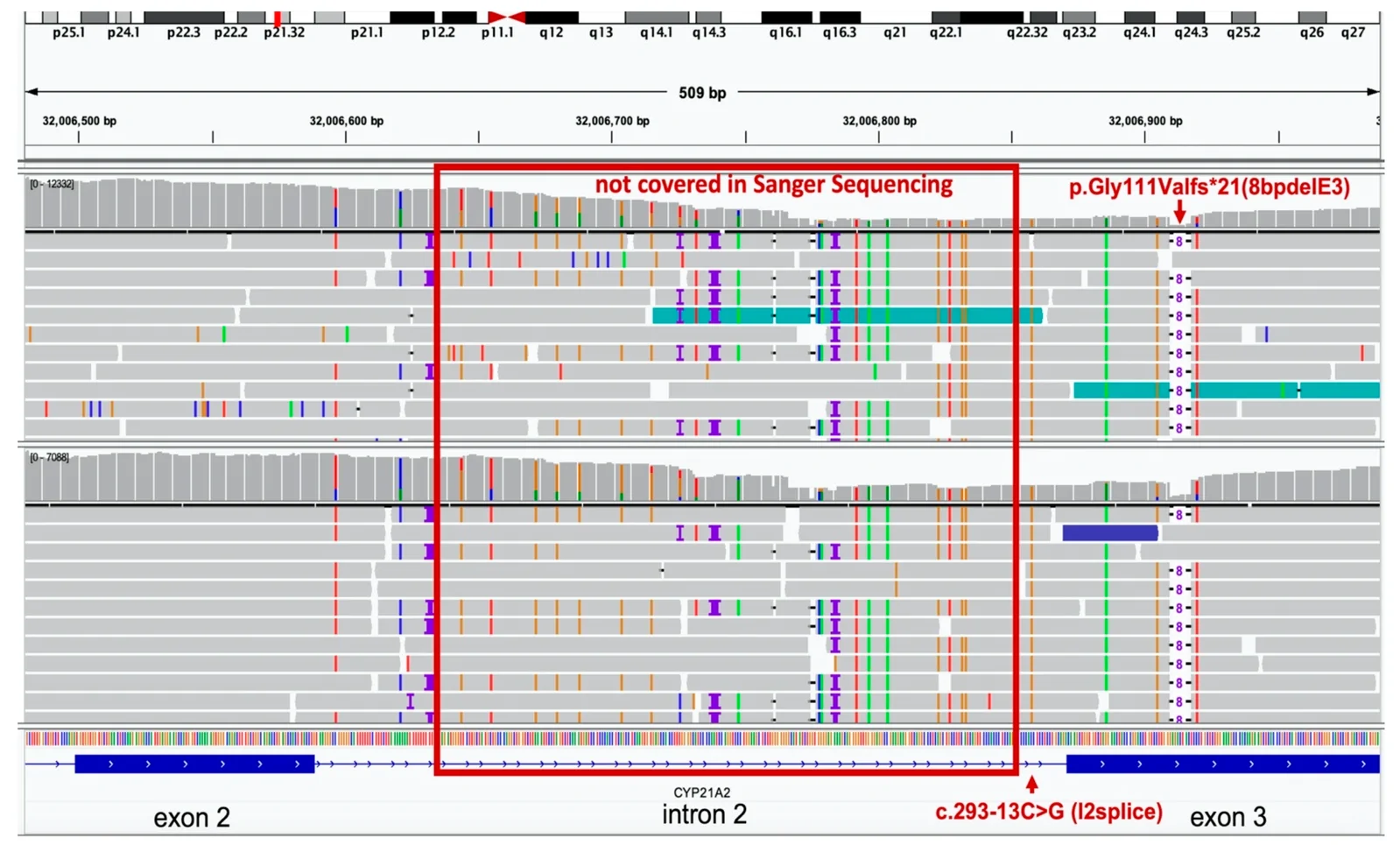
BAM files of sample 16 in intron 2 of the *CYP21A2* gene. The first BAM file represents the Sample 16 that underwent SR-NGS with the first approach (3 amplicons); the second BAM file is also sample 16 that underwent SR-NGS using the second approach (2 amplicons). The red box area in intron 2 was not covered by Sanger sequencing.

The SR-NGS results obtained from the pilot group were compared with those generated by Sanger sequencing, with discrepancies observed in eight samples (Table 2). These were attributed either to limitations of the bioinformatics pipeline or limitations of the Sanger sequencing approach. The only bioinformatics-related discrepancy involved VarScan mpileup2cns, which failed to call the pathogenic variant c.293-13C>G (legacy name: I2splice), while correctly reporting the benign heterozygous variant c.293-13C>A at the same genomic position (sample 4). Manual inspection of the BAM file confirmed the presence of both variants. Subsequent bioinformatics analysis using GATK HaplotypeCaller correctly called the heterozygous pathogenic variant c.293-13C>G alongside the benign variant c.293-13C>A in the same position. Limitations in the laboratory pipeline include discrepancies in eight samples, all of which were identified by SR-NG. In sample 15, SR-NGS identified the pathogenic variant p.Ile173Asn and three additional heterozygous benign variants that were initially missed by Sanger sequencing. These variants, with allele fractions ranging from 0.19 to 0.27, were subsequently confirmed through PCR amplification and Sanger sequencing using alternative internal primers targeting the corresponding *CYP21A2* region. In samples 16, 1, 2, 20, 21, 14 and 4, a lack of coverage in Sanger sequencing was observed at certain loci. More precisely, in sample 16, which harbored the CYP21A1P–CYP21A2 CH-1 chimeric form, SR-NGS detected all the anticipated intron 2 variants. However, comparison of the identified variants between SR-NGS and Sanger sequencing revealed that several benign variants were not identified by the latter in the 5′ UTR (positions NG_007941.3: g.4315–g.4675) and intron 2 (positions c.292+45–c.293-16) (Figure 4). This discrepancy was attributed to a lack of coverage at specific loci by Sanger sequencing. A similar observation was made in five additional samples harboring benign 5′ UTR variants, which were detected by SR-NGS but missed by Sanger sequencing (Table 2).

Comparison of the two variant callers showed high concordance. The total number of variants identified in the vcf files generated by GATK HaplotypeCaller and VarScan mpileup2cns was highly comparable, with only minor differences observed, mainly attributed to rare cases of alternative variant representation at the same genomic position, as observed for the c.293-13C>G and c.293-13C>A variants. Notably, GATK HaplotypeCaller identified all clinically relevant variants included in the pilot group, whereas VarScan mpileup2cns missed one pathogenic variant, c.293-13C>G.

Using Varscan mpileup2cns, specificity was 100% (95% CI: 99.99–100%) and sensitivity was 99.68% (95% CI: 98.23–99.99%) (Table 3). Using GATK HaplotypeCaller, specificity was 100% (95% CI: 99.99–100%) and sensitivity was 100% (95% CI: 98.83–100%) (Table 4). Its reproducibility and repeatability, including between-run variability, were also confirmed.

No differences in the identified variants between the first approach (three amplicons), and the second approach (two amplicons) were observed in the three samples of the pilot group analyzed by SR-NGS.

The SR-NGS assay could only detect small deletions/insertions, as shown in Figure 4. Deletions that involved the entire *CYP21A2* gene could not be detected. Furthermore, no significant difference was observed in the variant allele fraction of samples carrying duplications of the gene and samples carrying two copies of the gene.

### 3.3. SR-NGS Turnaround Time and Cost Evaluation

The laboratory procedure for each run (20 samples), from the PCR purification to loading on the MiSeq Sequencing System, had a turnaround time of 15 h. The bioinformatics pipeline and the analysis of the SR-NGS data for each run had a turnaround time of 24 h. Overall, the turnaround time from DNA extraction to the writing of the report was two weeks for 20 samples.

The cost for Sanger sequencing was 216.9€, whereas, for SR-NGS, was 119.8€ (excluding VAT).

### 3.4. NGS Results from the Study Group

In subgroup (a), 15.4% (28/182) of cases harbored pathogenic variants in homozygosity or compound heterozygosity, 35.7% (65/182) of cases harbored a pathogenic variant in heterozygosity, and in 42.9% (78/182) of cases, no pathogenic variant was detected (Table 5 and Table 6). VUS were identified in 1.6% of cases in heterozygosity and 1.6% of cases in compound heterozygosity with another pathogenic variant (Table 5 and Table 6). Duplications (with or without the p.Gln319Ter) *in trans* or not with a pathogenic variant were identified in 11.1% (5/45) of subjects tested (Table 7). All patients (28/28) were genetically diagnosed with the NC of 21-OHD.

In subgroup (c), 25% (4/16) of cases harbored a pathogenic variant of the NC; in 62.5% of cases, no pathogenic variant was identified; and 20% (2/10) of subjects tested harbored duplication of the gene (Table 5, Table 6 and Table 7).

In subgroup (b), the genetic diagnosis of the two patients confirmed the clinical diagnosis of 21-OHD.

**Table 5:** Pathogenic variants, VUS and deletions of the *CYP21A2* gene identified in heterozygotes of subgroups (a) and (c). Variants are presented at DNA and protein levels (where possible).

Heterozygotes (*n* = 72)			
Protein Change	Nucleotide Change	Number of Cases	Phenotype
**Subgroup (a)**			
-	c.[-126C>T;-113G>A;-110T>C;-103A>G;92C>T;293-13C>G;332_339del]	2	SW
-	c.[-126C>T;-113G>A;-110T>C;92C>T]	1	SV/NC
-	c.[-126C>T;-113G>A;-110T>C]	1	NC
-	c.-121C>T	1	-
-	c.[-113G>A;-110T>C;-103A>G]	1	NC
-	c.-115G>T	1	-
p.Pro31Leu	c.92C>T	2	SV/NC
-	c.293-13C>G	4	SW
p.Ile173Asn	c.518T>A	3	SV
p.Val282Leu	c.844G>T	34	NC
p.[Val282Leu;Leu308PhefsTer6;Gln319Ter;Arg357Trp]	c.[844G>T;923dup;955C>T;1069C>T]	1	SW
p.Leu289Phe	c.865C>T	1	-
p.Val305Met	c.913G>A	1	NC
p.Gln319Ter	c.955C>T	1	SW
p.Pro454Ser	c.1360C>T	7	NC
p.Pro483Ser	c.1447C>T	1	NC
-	c.*13G>A	5	Mild NC
-	c.[(?_-163)_(939+39_?)del]	1	SW
**Subgroup (c)**			
p.Val282Leu	c.844G>T	3	NC
-	c.*13G>A	1	Mild NC

The c.[-126C>T;-113G>A;-110T>C;-103A>G;92C>T;293-13C>G] corresponds to the chimeric CYP21A1P/CYP21A2 form CH-1. In the phenotype section, symbol - indicates that the effect on the phenotype is not known.

**Table 6:** Genotypes identified in compound heterozygotes/homozygotes of subgroup (a) and subgroup (b). VUS, pathogenic variants, as well as deletions of the gene are presented at DNA and protein levels (where possible).

Compound Heterozygotes/Homozygotes (*n* = 33)			
Protein Change	Nucleotide Change	Number of Cases	Phenotype
**Subgroup (** **b)**			
-	c.[293-13C>G];[293-13C>G]	1	SW
-	c.[293-13C>G];[955C>T]	1	SW
**Subgroup (a)**			
-	c.[-126C>T;-113G>A;-110T>C;-103A>G;92C>T;293-13C>G;332_339del];[92C>T]	1	SV/NC
-	c.[*13G>A];[-126C>T;-113G>A;-110T>C]	1	NC
-	c.[-115G>T];[844G>T]	1	-
-	c.[-115G>T];[1447C>T]	1	-
-	c.[92C>T];[*13G>A]	1	NC
p.[Pro31Leu];[Val282Leu]	c.[92C>T];[844G>T]	2	NC
-	c.[293-13C>G];[844G>T]	2	NC
-	c.[293-13C>G;1360C>T];[1447C>T]	1	NC
p.[Gly111ValfsTer21];[p.Val282Leu]	c.[332_339del];[844G>T]	1	NC
p.[Ile173Asn];[Val282Leu]	c.[518T>A];[844G>T]	2	NC
p.[Val282Leu];[Val282Leu]	c.[844G>T];[c.844G>T]	7	NC
p.[Val282Leu];[Pro483Ser]	c.[844G>T];[1447C>T]	2	NC
p.[Val282Leu];[Gln319Ter;Arg357Trp]	c.[844G>T];[955C>T;1069C>T]	1	NC
-	c.[844G>T];[*13G>A]	2	NC
p.[Val282Leu];[Gln319Ter]	c.[844G>T];[955C>T]	1	NC
p.[Leu289Phe];[Val282Leu]	c.[865C>T];[844G>T]	1	-
p.[Val282Leu];[Pro454Ser]	c.[844G>T];[1360C>T]	1	NC
-	c.[(?_-163)_(939+39_?)del];[844G>T]	1	NC
p.[Pro454Ser];[Pro483Ser]	c.[1360C>T];[1447C>T]	1	NC
p.[Arg357Trp];[Pro454Ser]	c.[1069C>T];[1360C>T]	1	NC

The c.[-126C>T;-113G>A;-110T>C;-103A>G;92C>T;293-13C>G] corresponds to the chimeric CYP21A1P/CYP21A2 form CH-1. In compound heterozygotes, carrying a VUS and a pathogenic variant, the effect on the phenotype is not known and as such, the symbol - is used.

**Table 7:** Genotypes of the study group subjects carrying duplication of the *CYP21A2* [with or without the p.Gln319Ter (c.955C>T)].

Second Allele	Allele Carrying Duplication of the *CYP21A2* Gene	Number of Cases
**Subgroup (a)**		
No pathogenic variant identified	c.[(?_-163)_(939+39_?)dup; 955C>T]	2
No pathogenic variant identified	c.[(?_-163)_(939+39_?)dup]	1
c.92C>T	c.[(?_-163)_(939+39_?)dup; 955C>T]	1
c.844G>T	c.[(?_-163)_(939+39_?)dup; 955C>T]	1
**Subgroup (c)**		
No pathogenic variant identified	c.[(?_-163)_(939+39_?)dup]	2

The c.92C>T and c.844G>T correspond to the p.Pro31Leu and p.Val282Leu respectively.

## 4. Discussion

This study describes a comprehensive pipeline for the use of SR-NGS assay in a diagnostic *CYP21A2* genotyping, highlighting its benefits and limitations after assessing its sensitivity and specificity when compared to Sanger sequencing.

In the laboratory pipeline, the discrepancies of the reported variants by SR-NGS and Sanger sequencing can be attributed to the limitations of the latter and, specifically, the lack of coverage in specific loci. More precisely, in sample 16, benign variants identified by SR-NGS (previously reported by Vrzalova et al. [26]) were not covered by Sanger sequencing, likely due to the initial primer design. The specific locus in intron 2 (position c.292+46 to c.293-20), in cases presenting with the CH-1 form, harbors several small (1–3 bp) deletions/insertions in heterozygosity, rendering sequencing of this region challenging. Although Sanger sequencing could not verify these variants, their existence is corroborated by the literature. In addition, the initial primer design for Sanger sequencing in the 5′ UTR does not always cover that loci (NG_007941.3:g.4434–4675) due to the presence of the benign variant c.16_18del in exon 1, preventing confirmation of their presence. In sample 15, Sanger sequencing failed to detect all variants identified by SR-NGS due to allele dropout. One of the PCR primers was designed at a locus where the cluster of three missense pathogenic variants is present in the pseudogene-p.[Ile237Asn;Val238Glu;Met240Lys]. As the subject harbored these variants, the allele dropout resulted in detection failure of those four variants that were successfully identified by SR-NGS. The initial design of the amplicons for SR-NGS as well as Sanger sequencing relies on the amplification of the whole *CYP21A2* gene in two or three specific overlapping fragments. This is a well-known strategy, but creates, as observed, the risk of allele dropout in Sanger sequencing. This could be avoided by applying the design proposed by Concolino et al., that relies on the amplification of the whole *CYP21A2* gene downstream of the *TNXB* (PCR product of 8.5 kb) [27]. Such design could also be integrated in this SR-NGS assay.

This study provides two independent variant-calling pipelines for their use in the *CYP21A2* diagnostic genotyping. These pipelines were also evaluated in order to be applied in a diagnostic setting. Their comparison revealed that the GATK HaplotypeCaller algorithm showed higher analytical accuracy and sensitivity for *CYP21A2* genotyping. This difference reflects the haplotype-based strategy implemented by GATK, which performs local de novo assembly and can accurately resolve complex variant patterns, including the presence of multiple variants at the same genomic position, as observed in the case of variant c.293-13. In contrast, Varscan mpileup2cns, which relies on pileup-based per-position allele counting, demonstrated reduced resolution in regions containing overlapping or closely spaced variants, potentially leading to incomplete variant detection. In addition, variant calling with Varscan mpileup2cns required a longer computational processing time as the generation of intermediate pileup files is a mandatory prerequisite for analysis, whereas GATK HaplotypeCaller operates directly on aligned BAM files. Nevertheless, both pipelines were deemed readily implementable and suitable for diagnostic *CYP21A2* analysis when adequate sequencing depth is achieved; however, when using Varscan mpileup2cns, manual inspection of BAM files remains essential to ensure correct interpretation and to avoid misdiagnosis at loci where multiple variants may coexist, such as in the case of the c.293-13. To our knowledge, this is the first report describing two different bioinformatics variant-calling pipelines for the analysis of the *CYP21A2* gene in a diagnostic setting *CYP21A2*.

Overall, the implementation of this SR-NGS assay has two primary limitations. First, it cannot determine the exact copy number of the *CYP21A2* gene, making MLPA analysis mandatory, particularly in cases where the p.Gln319Ter variant is present. This limitation can be justified by the substrate used, which, in this case, are PCR amplicons that are subject to several disadvantages such as PCR bias. However, it should be stated that algorithms for alterations in the copy number of the *CYP21A2* gene have not been tested. Second, the assay does not provide haplotype phasing (except for variants located in proximity), so parental samples are required for segregation analysis. Additionally, it should be noted that this assay cannot be used for the detection of complex genotypes that require extended protocols [27]. Nevertheless, some chimeric forms (such as CH-1) can be detected through the identified variants (multiple pathogenic variants with the presence of several benign variants that are usually identified in the pseudogene) but cannot provide the exact genomic rearrangement.

On the contrary, the advantage of this procedure is the simultaneous sequencing of up to 24 samples covering the whole *CYP21A2* gene (length: 3712 bp), rendering the SR-NGS assay rapid and easy to use. It should also be noted that the number of PCR reactions applied in the diagnostic *CYP21A2* SR-NGS assay does not affect its performance, indicating that laboratories with an existing design for the *CYP21A2* gene do not need to proceed to make any further changes in their workflow. Furthermore, this assay is cost-effective when compared to Sanger sequencing. Additionally, SR-NGS exhibits a higher sensitivity and specificity than Sanger sequencing, as already shown by this study, since it provides coverage of the whole *CYP21A2* gene and minimizes the likelihood of allele dropout. It should also be noted that this protocol is potentially adaptable in other platforms with minor modifications, but validation is still required. Hence, the SR-NGS assay can replace Sanger sequencing and, along with MLPA, can be widely used in a diagnostic setting for *CYP21A2* genotyping.

Moreover, applications of this SR-NGS assay can be expanded to genotyping other genes that harbor pseudogenes or homologous genes (such as the *CYP11B1* gene) or to combine more than one gene (gene panel) in the same assay with minor modifications in the laboratory and bioinformatics pipeline. Nevertheless, in such applications, validation is still mandatory.

In this study, the SR-NGS was diagnostically employed for the molecular investigation of the *CYP21A2* gene in the presented cohort. This cohort was categorized in three subgroups. In subgroup (a), all patients presented with the NC form of 21-OHD, which is in concordance with a previous study conducted by our center [14]. In cases that were found to harbor VUS in heterozygosity or compound heterozygosity with a pathogenic variant, reevaluation of the identified VUS was recommended after a period of two years. The number of heterozygotes identified in subgroup (c) are not representative since this subgroup has a limited size (*n* = 16).

Duplications, with or without the p.Gln319Ter pathogenic variant, were identified in 11.1% (5/45) and 20% (2/10) of subjects undergoing MLPA in subgroup (a) and subgroup (c) respectively. The high percentage of duplications in subgroups (a) and (c) is not indicative since the sample size of subjects tested in both subgroups is small. Nevertheless, not all negatively genotyped cases in subgroups (a) and (c) underwent MLPA analysis and, as a result, the percentage of duplications could be higher than the one reported in this study. However, it has been reported that duplications of the *CYP21A2* gene with no identified pathogenic variants are considered to exhibit normal function [6].

Recently, several protocols utilizing Long-Read Sequencing (LRS) have been applied [28,29,30,31,32,33], not only for the *CYP21A2* gene but also for other CAH genes. Although LRS is not yet widely used in diagnosis due to its high cost and error rate, efforts have been made to reduce these limitations [29,34]. Nevertheless, further studies are required to implement these protocols in the *CYP21A2* diagnostic genotyping.

In conclusion, to our knowledge, this is the first report of a comprehensive protocol for the use of SR-NGS in diagnostic *CYP21A2* genotyping, presenting the validation of an existing SR-NGS assay and the use of two assessed in-house bioinformatics pipelines, while outlining its benefits and limitations.

## Figures and Tables

**Table 1:** Pathogenic variants in the pilot group subjects.

Compound Heterozygotes (*n* = 8)	Heterozygotes (*n* = 7)
(1) c.[92C>T;293-13C>G];[1360C>T]	(12) c.518T>A (p.Ile173Asn)
(2) c.[68G>A];[317C>T;*13G>A]	
(3) c.[923dupT];[(?_-163)_(939+39_?)del]	(13) c.[844G>T;923dupT;955C>T;1069C>T] (p.[Val282Leu;Leu308PhefsTer6;Gln319Ter;Arg357Trp])
4) c.[92C>T];[293-13C>G]	(14) c.[-126C>T;-113G>A;-110T>C;-103A>G]
(5) c.[92C>T];[332_339del] (p.[Pro31Leu];[Gly111ValfsTer21])	(15) c.[710T>A;713T>A;719T>A;844G>T;923dupT] (p.[Ile237Asn;Val238Glu;Met240Lys;Val282Leu;Leu308PhefsTer6])
(6) c.[332_339del];[518T>A] (p.[Gly111ValfsTer21];[Ile173Asn])	(16 *) c.[-126C>T;-113G>A;-110T>C;-103A>G; 92C>T;293-13C>G; 332_339del]
(7) c.[518T>A];[844G>T] (p.[Ile173Asn];[Val282Leu])	(17) c.955C>T (p.Gln319Ter)
(8) c.[1439G>T];[1447C>T] (p.[Arg480Leu];[Pro483Ser])	(18) c.68G>A (p.Trp23Ter)
**Homozygotes (n = 3)**	**Heterozygote harboring duplication of the** ***CYP21A2*** **gene** ***in cis*** **with the c.955C>T (p.Gln319Ter)**
(9) c.[518T>A];[518T>A] (p.[Ile173Asn];[Ile173Asn])	
(10) c.[293-13C>G];[293-13C>G]	(19) c.[844G>T];[(?_-163)_(939+39_?)dup; 955C>T]
(11) c.[844G>T];[844G>T] (p.[Val282Leu];[Val282Leu]	**Negative genotyped cases (n = 2, samples 20 and 21)**

* Sample 16 harbors the CYP21A1P-CYP21A2 CH1 chimeric form. In compound heterozygotes/homozygotes, the genotypes are presented at the DNA level followed by the protein level (where possible) in parenthesis. In heterozygotes, the pathogenic variants are presented at the DNA level followed by the protein level (where possible) in parenthesis.

**Table 2:** Discrepancies observed in the samples of the pilot group.

**Sample Number**	**Bioinformatics Pipeline**		**Explanation**	**Action**
	**Varscan mpileup2cns (v2.4.6)**	**Sanger Sequencing**		
4	X	✓	Failure to call the variant c.293-13C>G from the vcf file using Varscan mpileup2cns.	GATK HaplotypeCaller was used.
**Sample Number**	**Identification of Variants**		**Explanation**	**Action**
	**SR-NGS**	**Sanger sequencing**		
4	✓	X	The variant NG_007941.3:g.4568A>G was not identified in Sanger sequencing.	No action required.
15	✓	X	Failure due to allele dropout. The pathogenic variant p.Ile173Asn and variants c.550-15C>A, c.550-8T>C and p.Asp184Glu were absent Sanger sequencing.	Alternative primers were used in Sanger sequencing as to verify the presence or absence of those variants.
16	✓	X	Absence of coverage in the initial design.	No action required.
1	✓	X	The variant NG_007941.3: g.4434G>A was not identified in Sanger sequencing.	No action required.
2	✓	X	The variant NG_007941.3:g.4583C>T was not identified in Sanger sequencing.	No action required.
20	✓	X	The variant NG_007941.3:g.4583C>T was not identified in Sanger sequencing. Absence of coverage in the initial design.	No action required.
21	✓	X	The variant NG_007941.3:g.4583C>T was not identified in Sanger sequencing.	No action required.
14	✓	X	The variant NG_007941.3:g.4675dup was not identified in Sanger sequencing.	No action required.

✓ indicates that the method identified/called all variants; X: indicates that the method did not identify or call all the variants.

**Table 3:** Specificity sensitivity and confidence intervals for each sample with Varscan mpileup2cns.

Samples	TP	FN	FP	TN	Total Evaluable (TP, FN, FP, TN)	Sensitivity Report	Specificity Report
1	14	0	0	3242	3256	100% (76.8–100%)	100% (99.9–100%)
2	16	0	0	3263	3279	100% (79.4–100%)	100% (99.9–100%)
3	10	0	0	3454	3464	100% (69.2–100%)	100% (99.9–100%)
4	16	1	0	3262	3279	94.1% (71.3–99.9%)	100% (99.9–100%)
5	9	0	0	1692	1701	100% (66.4–100%)	100% (99.8–100%)
6	15	0	0	3448	3463	100% (78.2–100%)	100% (99.9–100%)
7	16	0	0	3448	3464	100% (79.4–100%)	100% (99.9–100%)
8	14	0	0	3452	3466	100% (76.8–100%)	100% (99.9–100%)
9	15	0	0	3448	3463	100% (78.2–100%)	100% (99.9–100%)
10	12	0	0	3457	3469	100% (73.5–100%)	100% (99.9–100%)
11	10	0	0	3454	3464	100% (69.2–100%)	100% (99.9–100%)
12	13	0	0	3456	3469	100% (75.3–100%)	100% (99.9–100%)
13	15	0	0	3264	3279	100% (78.2–100%)	100% (99.9–100%)
14	22	0	0	3048	3070	100% (84.6–100%)	100% (99.9–100%)
15	22	0	0	3441	3463	100% (84.6–100%)	100% (99.9–100%)
16	28	0	0	3247	3275	100% (87.7–100%)	100% (99.9–100%)
17	14	0	0	3452	3466	100% (76.8–100%)	100% (99.9–100%)
18	12	0	0	3454	3466	100% (73.5–100%)	100% (99.9–100%)
19	17	0	0	3449	3466	100% (80.5–100%)	100% (99.9–100%)
20	12	0	0	3267	3279	100% (73.5–100%)	100% (99.9–100%)
21	10	0	0	3269	3279	100% (69.2–100%)	100% (99.9–100%)

**Table 4:** Specificity, sensitivity and confidence intervals for each sample with GATK HaplotypeCaller.

Samples	TP	FN	FP	TN	Total Evaluable (TP, FN, FP, TN)	Sensitivity Report	Specificity Report
1	14	0	0	3242	3256	100% (76.8–100%)	100% (99.9–100%)
2	16	0	0	3263	3279	100% (79.4–100%)	100% (99.9–100%)
3	10	0	0	3454	3464	100% (69.2–100%)	100% (99.9–100%)
4	17	0	0	3262	3279	100% (80.5–100%)	100% (99.9–100%)
5	9	0	0	1692	1701	100% (66.4–100%)	100% (99.8–100%)
6	15	0	0	3448	3463	100% (78.2–100%)	100% (99.9–100%)
7	16	0	0	3448	3464	100% (79.4–100%)	100% (99.9–100%)
8	14	0	0	3452	3466	100% (76.8–100%)	100% (99.9–100%)
9	15	0	0	3448	3463	100% (78.2–100%)	100% (99.9–100%)
10	12	0	0	3457	3469	100% (73.5–100%)	100% (99.9–100%)
11	10	0	0	3454	3464	100% (69.2–100%)	100% (99.9–100%)
12	13	0	0	3456	3469	100% (75.3–100%)	100% (99.9–100%)
13	15	0	0	3264	3279	100% (78.2–100%)	100% (99.9–100%)
14	22	0	0	3048	3070	100% (84.6–100%)	100% (99.9–100%)
15	22	0	0	3441	3463	100% (84.6–100%)	100% (99.9–100%)
16	28	0	0	3247	3275	100% (87.7–100%)	100% (99.9–100%)
17	14	0	0	3452	3466	100% (76.8–100%)	100% (99.9–100%)
18	12	0	0	3454	3466	100% (73.5–100%)	100% (99.9–100%)
19	17	0	0	3449	3466	100% (80.5–100%)	100% (99.9–100%)
20	12	0	0	3267	3279	100% (73.5–100%)	100% (99.9–100%)
21	10	0	0	3269	3279	100% (69.2–100%)	100% (99.9–100%)

## Data Availability

The data presented in this study are available on request from the corresponding author due to privacy/ethical reasons.

## Abbreviations

The following abbreviations are used in this manuscript:

SR-NGS	Short Read Next-Generation Sequencing
21-OHD	21-Hydroxylase Deficiency
CAH	Congenital Adrenal Hyperplasia
EMQN	European Molecular Genetics Quality Network
BAM	Binary Format
VUS	Variant of Uncertain Significance
LRS	Long-Read Sequencing
SAM	Sequence Alignment Map
MLPA	Multiplex Ligation-Dependent Probe Amplification

## Appendix A. Primer’s Sequences for Each Amplicon

**Amplicon**	**Forward Primer (5′–3′)**	**Reverse Primer (5′–3′)**
Amplicon 1	TCA AAC CAG CTC AAG GTG GG	CAG AGC AGG GAG TAG TCT C
Amplicon 2	TCA AAC CAG CTC AAG GTG GG	CAG CTG CAT CTC CAC GAT G
Amplicon 3	CCT GTC CTT GGG AGA CTA CT	TCT CGC ACC CCA GTA TGA CT
